# Entomological indicators of *Plasmodium* species transmission in Goma Tsé-Tsé and Madibou districts, in the Republic of Congo

**DOI:** 10.1186/s12936-023-04823-9

**Published:** 2024-01-16

**Authors:** Jacques Dollon Mbama Ntabi, Abel Lissom, Jean Claude Djontu, Francis N. Nkemngo, Steve Diafouka-Kietela, Jolivet Mayela, Georges Missontsa, Luc Djogbenou, Cyrille Ndo, Charles Wondji, Ayola Akim Adegnika, Arsène Lenga, Steffen Borrmann, Francine Ntoumi

**Affiliations:** 1 Fondation Congolaise Pour La Recherche Médicale, Brazzaville, Republic of the Congo; 2 Faculté Des Sciences Et Techniques, Université Marien Ngouabi, Brazzaville, Republic of the Congo; 3 Institute of Tropical Medicine, University of Tübingen, Tübingen, Germany; 4 Department of Biological Science, Faculty of Science, University of Bamenda, Bamenda, Cameroon; 5 Department of Parasitology and Medical Entomology, Centre for Research in Infectious Diseases (CRID), Centre Region, Yaounde, Cameroon; 6 Tropical Infectious Deseases Research Center (TIDRC), University of Abomey-Calavi, Cotonou, Benin; 7 Department of Parasitology and Microbiology, Center for Research in Infectious Diseases (CRID), Yaoundé, Cameroon; 8 Department of Vector Biology, Liverpool School of Tropical Medicine, Pembroke Place, Liverpool, L3 5QA UK; 9 Department of Biological Sciences, Faculty of Medicine and Pharmaceutical Sciences, University of Douala, Douala, Cameroun; 10 Centre de Recherches Médicales de Lambaréné, Lambaréné, Gabon; 11 German Center of Infection Research (DZIF), Tübingen, Germany

**Keywords:** Malaria, *Anopheles* vectors, Transmission, Rural and urban areas, Republic of Congo

## Abstract

**Background:**

Malaria remains a major public health problem in the Republic of Congo, with *Plasmodium falciparum* being the deadliest species of *Plasmodium* in humans. Vector transmission of malaria is poorly studied in the country and no previous report compared rural and urban data. This study aimed to determine the *Anopheles* fauna and the entomological indices of malaria transmission in the rural and urban areas in the south of Brazzaville, and beyond.

**Methods:**

Indoor household mosquitoes capture using electric aspirator was performed in rural and urban areas during raining and dry seasons in 2021. The identification of *Anopheles* species was done using binocular magnifier and nested-PCR. TaqMan and nested-PCR were used to detect the *Plasmodium* species in the head/thorax and abdomens of *Anopheles*. Some entomological indices including the sporozoite infection rate, the entomological inoculation rate and the man biting rate were estimated.

**Results:**

A total of 699 *Anopheles* mosquitoes were collected: *Anopheles gambiae *sensu lato (*s.l*.) (90.7%), *Anopheles funestus s.l.* (6.9%), and *Anopheles moucheti* (2.4%). Three species of *An. gambiae s.l*. were identified including *Anopheles gambiae *sensu stricto (78.9%), *Anopheles coluzzii* (15.4%) and *Anopheles arabiensis* (5.7%). The overall sporozoite infection rate was 22.3% with a predominance of *Plasmodium falciparum*, followed by *Plasmodium malariae* and *Plasmodium ovale*. *Anopheles* aggressiveness rate was higher in households from rural area (1.1 bites/night) compared to that from urban area (0.8 ib/p/n). The overall entomological inoculation rate was 0.13 ib/p/n. This index was 0.17 ib/p/n and 0.092 ib/p/n in rural and in urban area, respectively, and was similar during the dry (0.18 ib/p/n) and rainy (0.14 ib/p/n) seasons.

**Conclusion:**

These findings highlight that malaria transmission remains high in rural and urban area in the south of Republic of Congo despite the ongoing control efforts, thereby indicating the need for more robust interventions.

## Background

Malaria remains a major public health problem worldwide, with 247 million cases reported in 2021 compared to 245 million cases in 2020 [1]. The estimated number of malaria deaths stood at 619,000 in 2021, compared to 625 000 in 2020 [1]. The sub-Saharan countries continue to carry the heaviest burden of the disease (about 95% of the cases and 96% of deaths) [1].

Malaria parasite is transmitted to people through the bites of infected female *Anopheles* mosquitoes [2]. A total of 484 *Anopheles* mosquitoes species have been described across the world, and 70 are known as malaria vectors, with only 30–40 species being able to transmit in Africa [2, 3]. Many factors have been reported to influence the epidemiology and severity of malaria disease in endemic setting, including the heterogeneity of the environment, the degradation of the forests, the extent of unplanned urbanization, the development of urban agriculture, and the degree of human migration from rural to urban areas [4–7].

In Central Africa, the diversity of mosquitoes and the malaria transmission dynamic have been widely studied, mainly in Cameroon, Gabon and the Democratic Republic of Congo (DRC) [6, 8]. In this Central Africa sub-region, about eighteen anopheline species have been reported to be involved in the transmission of *Plasmodium* parasites [2, 3]. This include major vector species, such as *Anopheles gambiae *sensu stricto (*s.s*.), *Anopheles coluzzii*, *Anopheles arabiensis*, *Anopheles funestus*, *Anopheles nili*, *Anopheles moucheti* which are known for their high anthropophilic behaviour [2, 3, 9]. Some of these vectors exhibit the ability to adapt to changing environmental conditions [10, 11], even though each of them shows ecological preferences.

The Republic of Congo is one of the 54 countries where malaria transmission remains worrisome. According to the report of the National Malaria Control Programme (NMCP), malaria is responsible for 63% of medical consultations, 20% of hospitalization rate and 9% of deaths in the country [12]. The transmission of the disease in the Republic of Congo is perennial, and differs between urban, peri-urban and rural areas, with *Plasmodium falciparum* as the major *Plasmodium* species [13, 14].

The latest data on the *Anopheles* fauna in the Republic of Congo dates back to 1985 and described the presence of seven species, *An. gambiae *sensu lato (*s.l.*), *An. funestus s.l., An. nili, An. moucheti, Anopheles paludis, Anopheles pharoensis* and *Anopheles hankocki*, with species of the *An. gambiae* group being the major vectors of malaria transmission during that period [15, 16]. The vector control of malaria in Congo relies particularly on the use of long-lasting insecticide-treated mosquito nets approved by the NMCP [13]. So far, there is a scarcity of data comparing the entomological indicators of the transmission of *Plasmodium* species infection in rural and urban setting in Republic of Congo. Basic information about the *Anopheles* fauna and the entomological indices of malaria transmission in the communities, are critical for planning and implementing anti-vector control measures in the country. The present study aimed to characterize the *Anopheles* fauna and determine the entomological indices of malaria transmission in the southern of Brazzaville and beyond.

## Methods

### Study sites

**Fig. 1:**
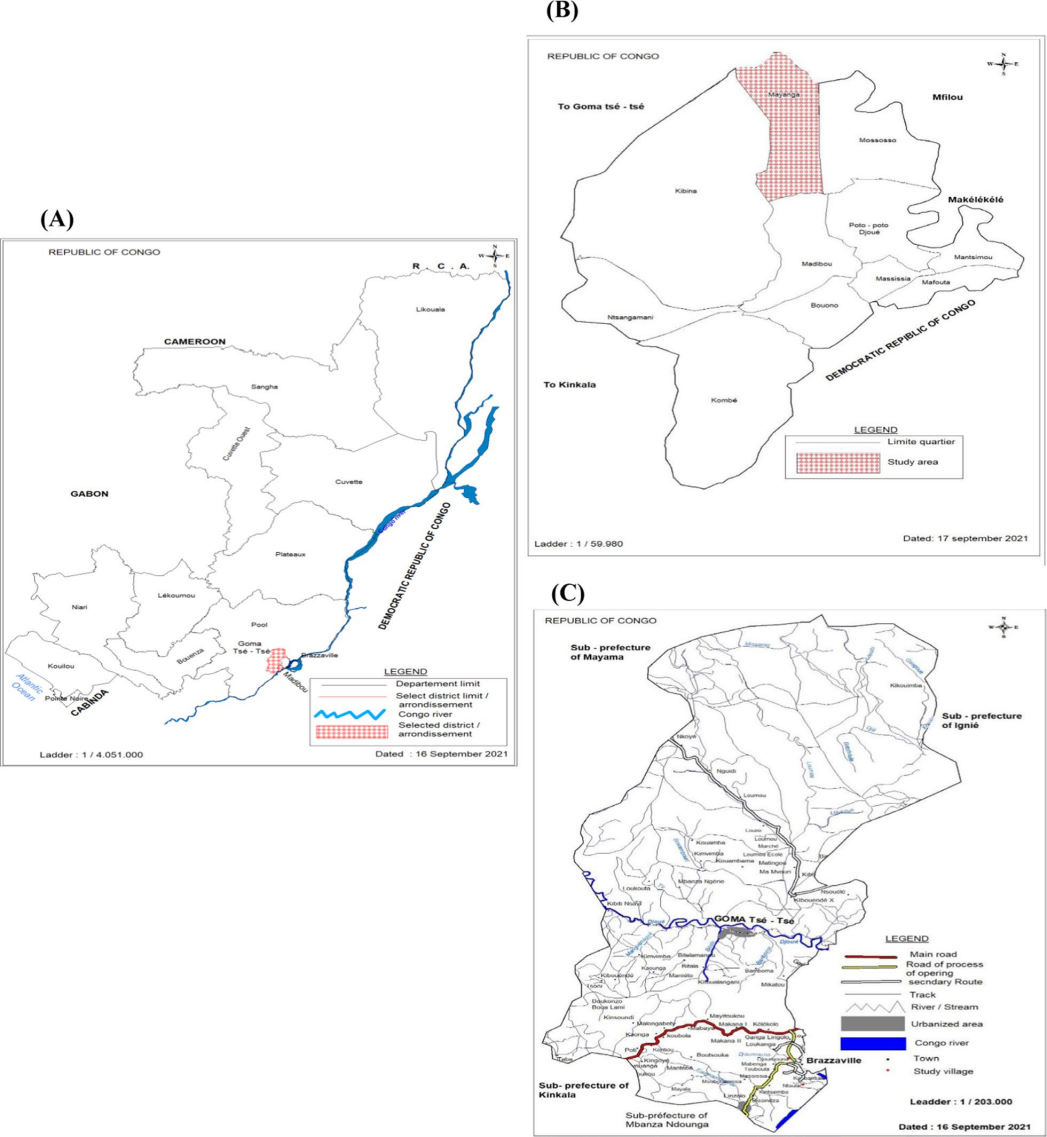
Map of Republic of Congo (**A**) showing localities surveyed in Goma Tsé-Tsé (**B**) and Madibou (**C**) district. In Goma Tsé-tsé district, the size of the red dot represents the selected village (Ntoula and Djoumouna), and in Madibou district the red striped area represents the selected city (Mayanga) for the survey

The study was conducted in the rural area of Pool (Goma Tsé-Tsé district: Ntoula and Djoumouna villages) and the urban area of Brazzaville (Mayanga) characterized by a major bio-ecological belts [13] (Fig. 1). The departments of Brazzaville and Pool are situated in the south of Republic of Congo. This part of the country have a tropical humid climate divided in two seasons: dry season (from June to September, and January to February) and rainy season (from October to January, and March to May). These study sites have different sociological and ecological characteristics which can be considered as representative regions of the Congolese savannah and degraded forest biotopes capable of sheltering the different species of the Culicidae mosquito family. The Goma Tsé-Tsé district is geo-localized at 4° 24′ 44" S, 15° 8′ 31" E and is situated at average altitude of 293 m. The Madibou district is localized between 4° 18′ 35" S, 15° 11′ 30" E [17]. The average humidity varies between 78 and 84%. Goma Tsé-Tsé district is located at about 15 km from Brazzaville in an area of degraded secondary forest and savannah. It is characterized by the presence of a gallery forest bordering the permanent Djoumouna river and Ntoula river [13]. This locality is surrounded by many rivers including Lomba, Kinkoue, Loumbangala, Djoumouna, Loumou and Congo rivers which supply to a series of fish farming ponds [18] that can serve as the potential foci of malaria vectors. The main activities in this area are farming and fishing. Mayanga is an urban area of the Madibou district situated in the south of Brazzaville. It is characterized by the presence of three site of market gardening (Agri-Congo 1 and 2 and the Groupement Jean Felicien Mahouna), and irrigated by three rivers including Djoué, Laba and Matou rivers [13]. Mayanga accounts several public and private service such as the health centres, the primary and high schools.

### Study design and adult mosquito collection

A cross-sectional survey study was carried out during raining and dry seasons, from March to September 2021. The mosquitoes were collected every morning (5:00 am to 10:00 am) in the households of the participants after obtaining their informed consent form. The indoor capture of mosquitoes resting on the walls and roofs of houses was undertaken using the electric aspirator as previously described (Rule In-Line Blowers, Model 240). The captured mosquitoes in each dwelling were placed in the cups with the code or the identification number of the house. The blood-fed mosquitoes were kept for two days in the cup before processing, in order to allow the complete digestion of human blood in their digestive tract. Data were registered in well-structured collection sheets including the district name, household identification number, type of house, number of bedrooms, and number of people living in the household, number of trapped mosquitoes, date and time of mosquitoes collection. The mosquitoes were then transported to the laboratory for analysis.

### Laboratory processing of mosquito samples

The morphological identification of *Anopheles* mosquitoes was done using the "LEICA ZOOM 2000" binocular magnifier at the magnitude of 40X, based on morphological criteria following the identification keys of Gillies and De Meillon [19] and Gillies and Coetzee [20]. Female *Anopheles* mosquitoes were sorted in Eppendorf tubes and transported to the medical entomology laboratory of CeRMI (Centre de Recherche sur les Maladies Infectieuses -Christophe Mérieux) for subsequent analyses. The females *Anopheles* were dissected in to Abdomens (Abd) and Heads/thoraces (H/T). Each part of *Anopheles* mosquito were submitted processed for detection of oocyst (Abd) and sporozoites (H/T) as previously described [21]. All samples were stored at −20 °C.

### Molecular characterisation of mosquito and Plasmodium infection detection

The DNA of heads/thorax and abdomens of all female *Anopheles* were extracted separately using the LIVAK method adapted for mosquito DNA extraction as previously described by Livak et al*.* [22].

### Identification of species of the An. gambiae complex and the An. funestus group

The *An. gambiae complex* and the *An. funestus* group were discriminated by PCR using the abdomens part of each mosquito. For *An. gambiae s.l*., primers targeting *SINE200* insertion (Short Interspersed Elements) was used to distinguish *An. gambiae s.s.* from *An. coluzzii* and *An. arabiensis* [23]. Primers targeting ITS2 region (Second Internal Transcribed Spacer Region) of nuclear ribosomal deoxyribonucleic acid (rDNA) was used to produce of varying band sizes to distinguish members of the *An. funestus* group [24]. The amplification reactions of *SINE200* fragments was carried out in the total volume of 15 µL including 1.5 µL of PCR buffer 10x, 0.75 µL of 25 mM MgCl_2_, 0.12 µL of 10 mM dNTPs, 0.51 µL of 10 µM SINE_Foward and 0.51 µL of 10 µM SINE_Reverse primers, 0.12 µL of Kapa *Taq* DNA polymerase 5U/µL, 10.49 µL of sigma free water and 1 µL DNA. The PCR device used for amplification was the thermocycler (Master X50a Eppendorf AG, Hamburg, Germany) and the amplification included: denaturation at 95 °C for 5 min, followed by 35 cycles of 30 s denaturation at 95 °C, 1 min annealing at 54 °C, 1 min extension at 72 °C, a final step of 10 min at 72 °C. For the ITS2 fragment, the PCR mix of 14 µL included 1.5 µL of PCR buffer 10x, 0.9 µL of 25 mM MgCl_2_, 0.12 µL of 10 mM dNTPs, 0.51 µL of 10 µM ITS2A, 0.51 µL of 10 µM ITS2B 0.51 µL of 10 µM FUN, 0.51 µL of 10 µM RIV, 0.51 µL of 10 µM PAR, 0.51 µL of 10 µM RIVLIK, 0.51 µL of 10 µM LEES, 0.51 µL of 10 µM VAN reverse primers, 0.12 µL of Kapa *Taq* DNA polymerase 5U/µL, and 7.28 µL of sigma free water. A volume of 1 μL of extracted DNA for each sample, was added in to the master mix, and the amplification carried out using the following conditions: initial denaturation for 5 min at 95 °C, followed by 35 cycles of 30 s denaturation at 95 °C, 1 min annealing at 50 °C, 1 min extension at 72 °C, final step of 10 min at 72 °C and a hold at 10 °C. After amplification, 6 μL of the PCR reaction was mixed with 4 μL of syber green and electrophoresed on a 2% agarose gel (2 g agarose in 100 ml of 89 mM Tris, 89 mM Boric acid, 2 mM EDTA), at a constant voltage of 80 V for 50 min. Both strands of the amplified fragments (*SINE200* and ITS2) were visualized on the GelDoc™ EZ Imager (Bio-Rad Laboratories, Hercules, CA, USA). The details of primers targeting *SINE200* and ITS2 of *An. gambiae s.l.* and *An. funestus* group are presented in Table 1.

**Table 1:** Primers using in molecular identification of *An. gambiae s.l.* in *Plasmodium* infection

	Primer	Primer sequences	Attending band
Primers	Plas_F	5ʹ-GCTTAGTTACGATTAATAGGAGTAGCTTG-3ʹ	
	Plas_R	GAAAATCTAAGAATTTCACCTCTGACA-3ʹ	
Probe	Falci +	5ʹ- TCTGAATACGAATGTC-3ʹ	FAM
	OVM +	5ʹ- CTGAATACAAATGCC-3ʹ	HEX
First round PCR	rPLU6	5ʹ-TTA AAA TTG TTG CAG TTA AAA CG-3ʹ	
	rPLU5	5ʹ-CCT GTT GTT GCC TTA AAC TTC-3ʹ	
Second round PCR	rFAL1	5ʹ-TTA AAC TGG TTT GGG AAA ACC AAA TAT ATT-3ʹ	205 bp
	rFAL2	5ʹ-ACA CAA TGA ACT CAA TCA TGA CTA CCC GTC-3ʹ	
	rMAL1	5ʹ-ATA ACA TAG TTG TAC GTT AAG AAT AAC CGC-3ʹ	105 bp
	rMAL2	5ʹ-AAA ATT CCC ATG CAT AAA AAA TTA TAC AAA-3ʹ	
	rOVA1	5ʹ-ATC TCT TTT GCT ATT TTT TAG TAT TGG AGA-3ʹ	800 bp
	rOVA2	5ʹ-GGA AAA GGA CAC ATT AAT TGT ATC CTA GTG-3ʹ	
	rVAV1	5ʹ-GCT TCG GCT TGG AAG TCC-3ʹ	120 bp
	rVAV2	5ʹ-CCG AAT TCA GTC CCA CGT-3ʹ	
SINE_200	Sine_F	TCG CCT TAG ACC TTG CGT TA	Ag ss = 249 bp Ac = 479, Aa = 223
	Sine_R	CGC TTC AAG AAT TCG AGA TAC	
COCTAIL *An. funestus* group multiplex PCR	ITS2A	5ʹ TGT GAA CTG CAG GAC ACA T 3ʹ	
	ITS2B	5ʹ TAT GCT TAA ATT CAG GGG GT 3ʹ	
	UV	5ʹ TGT GAA CTG CAG GAC ACA T 3ʹ	
	FUN	5ʹ GCA TCG ATG GGT TAA TCA TG 3ʹ	506
	VAN	5ʹ TGT CGA CTT GGT AGC CGA AC 3ʹ	578
	RIV	5ʹ CAA GCC GTT CGA CCC TGA TT 3ʹ	411
	PAR	5ʹ TGC GGT CCC AAG CTA GGT TC 3ʹ	252
	LEES	5ʹ TAC ACG GGC GCC ATG TAG TT 3ʹ	146

### Detection of Plasmodium sporozoites and oocysts in Anopheles mosquitoes

### TaqMan assay

The protocol described by Bass C, Nikou D, Blagborough AM and al. [25] was used for genus-specific amplification targeting the 18S rRNA genes of *Plasmodium* [25] through detecting the sporozoites in the salivary glands (heads/thorax) and the oocysts in the abdomens of female *Anopheles*. The PCR mix was carried out in a reaction volume of 10 µL comprising 1 µl of matrix DNA, 5 µL (1 µM) of SensiMix II Probe (1.25 ml), 0.8 µl (10 mM) of sense primers (PlasF: forward primer), 0.8 µl (10 mM) of antisense primers (PlasR: reverse primer), 0.3 µL of Falci + , 0.2 µL of OVM + and 1.9 µL of water. The amplification was carried out in the Light Cycler 480 real-time PCR system (Roche, SN: 20,726) using the following conditions: a pre-denaturation at 95 °C for 10 min, followed by 40 cycles of 15 s at 92 °C and 1 min at 60 °C.

The probe Falcip + was labelled with 6-FAM for the detection of *P. falciparum* and the probe OVM^+^ was labelled with HEX for the detection of *Plasmodium malariae*, *Plasmodium ovale* or *Plasmodium vivax* and primers, Plas_Foward and Plas_Reverse were used together with two probes tagged with fluorophores FAM and HEX. Known positive samples of *P. falciparum* and other *Plasmodium* species were used as positive controls and sterile water as negative control. All positive samples were subjected to 18 s Nested-PCR to confirm and discriminate *Plasmodium* species detected by TaqMan using the previously described protocol by Boonma P, Christensen PR, Suwanarusk R, Price RN, Russell R and Lek-Uthai U [26], with slight modification (use of Dream Taq instead of Taq polymerase) Nkemngo et al*.*[27]. The details of primers and probes are presented in the Table 1.

### Nested-PCR

Amplification targeting the 18S rRNA genes of *Plasmodium* was performed in two steps. The first PCR reaction was to amplify the portion of all *Plasmodium* genus in the 20 μl of total volume (12.5 µL of distilled water, 2.5 µL of PCR buffer X10, 1.25 µL of 25 mM MgCl_2,_ 0.5 µL of dNTPs 10 nM, 0.5 µL of 10 µM rPLU5 forward, 0.5 µL of 10 µM rPLU6 reverse primers, 2 µL of genomic DNA and 0.25 µL of 5U/µL Dream Taq DNA polymerase. The amplification included: denaturation at 94 °C for 4 min; followed by 35 cycles of denaturation at 94 °C for 30 s, annealing at 55 °C for 1 min and extension at 72 °C for 1 min, with a final extension at 72 °C for 4 min and the hold at 4 °C. The second PCR reaction was for the speciation of the malaria parasite using the product of first PCR reaction as template and the primers designed to amplify the specific sequences of *P. falciparum* (rFAL1/rFL2)*, P. ovale* (rOVA1/rOVA2)*, P. malariae* (rMAL1/rMAL2) and *P. vivax* (rVAV1/rVAV2) as presented in the Table 1. For this second PCR reaction, 1 μL of the product of the first PCR was added in 19 μL of master mix prepared as described above and amplified using the thermocycler (Master X50a Eppendorf AG, Hamburg, Germany under the same cycling conditions as described for the first PCR reaction, with the exception that the annealing temperature was 58 °C. Details of the primers used are provided in the Table 1.

### Data analysis

Data was entered using Microsoft Excel (Microsoft Inc., Redmond, WA, USA) version 2016. All statistical analyses were performed using GraphPad_Prism_6.01 software version 22.1 (SPSS, IBM Corp., Armonk, NY, USA) and processed by the Contingency statistical test. The prevalence of *Plasmodium* spp. infections in the *Anopheles* population was determined as the proportion of female *Anopheles* identified as positive for the presence of *Plasmodium* (either for all *Plasmodium* parasite species or for an individual species) at each site of study. Fisher exact test was used for the comparison of proportion in case of very low samples size for some groups (n < 5), while Chi-square test was used for the comparison of proportion between groups with sample size higher than 4 (n ≥). The significance level was set at P < 0.05.

**Entomological indexes** of malaria transmission included in this study are man biting Rate (ma), Infection Rate (s), Entomological Inoculation Rate (EIR).

**Man biting rate (ma)**: Also called aggressive density is the product of anopheline density in contact with humans (m) and the anthropophily rate (a). It is calculated by dividing the total number of engorged females **(F)** of a species captured by the total number of people **(W)** who spent the night in the rooms where the captures took place. The Aggression Rate is expressed as the number of *Anopheles* mosquito bites per man per night.

$$ma=F\div W$$

***Plasmodium* infection rate (s)** is the proportion of mosquitoes infected or carrying sporozoites in their salivary glands. This index is expressed as a percentage (number of infected mosquitoes out of the number of mosquitoes examined times hundred).

$$s=\frac{Number\,\,of\,\,mosquitoes\,positive}{Number\,\,of\,\,mosquitoes\,tested} X 100$$

**Entomological inoculation rate (EIR)** is the number of infective bites from *Anopheles* during a given period of time. It is expressed by infective bites per man per night/day/week/month or per year.

$$EIR=ma\times s$$

### Ethical considerations

This study received ethical approval from the Institutional Ethics Committee of Fondation Congolaise pour la Recherches Médicales (No. 013/CIE/FCRM/2018), administrative authorizations from Université Marien Ngouabi (UMNG) (No. 247/UMNG. FST.DFD.FD-SBIO) and each local authority of the study areas. An informed consent was signed by the head of each household, before any indoor household mosquito capture. Each household head was free to withdraw from the study at any time without any justification.

## Results

### Mosquito composition in the study sites

Out of 699 *Anopheles* mosquitoes captured in this study, *An. gambiae s.l.* (90.7%; 634/699) was the predominant species followed by *An. funestus s.l.* (6.9%; 48/699), and *An. moucheti* (2.4%; 17/699). The distribution of these mosquito species was similar in rural (90.5% *An. gambiae* s*.l.,* 6.9% *An. funestus s.l.*, and 2.6% *An. moucheti*) and urban (92.0% *An. gambiae s.l*., 6.9% *An. funestus s.l.*, and 1.1% *An. moucheti*) setting (Table 1). *Anopheles gambiae s.l*. was significantly (p < 0.0001) more abundant in dry season (95.6%; 301/315) compared to the rainy season (86.7%; 333/384), while it was the contrary for the distribution of *An. funestus s.l*. (10.7% in rainy vs 2.2% in dry seasons; p < 0.0001). The abundance of *An. moucheti* was similar, with 2.6% in rainy season and 2.2% in dry season (p = 0.8095). The molecular analysis of the 634 females of the Gambiae complex showed that *An. gambiae s.s.* was the predominant species (78.9%), followed by *An. coluzzii* (15.4%) and *An. arabiensis* (5.7%) (Table 2). The distribution of these species was similar among the seasons, while *An. gambiae s.s.* and *An. coluzzii* were predominant in urban (92.5%) and rural (17.3%) areas, respectively, compared to their counterparts (76.9% *An. gambiae s.s.* in rural; 2.5% *An. coluzzii* in urban). The molecular characterization of 48 specimens of *funestus* group showed only the presence of *An. funestus s.s.* (100%).

**Table 2:** Distribution of the *Anopheles* species/species complex (A) and the *An. gambiae s.l.* sibling species with respect to the study areas and the seasons

Species	Areas		p-value	Seasons		p-value
	Rural	Urban		Rainy	Dry	
Distribution of *Anopheles* species/species complex % (n)						
*An. gambiae s.l.* %	90.5 (554)	92.0 (80)	0.08437	86.7 (333)	95.6 (301)	< 0.0001
*An. funestus*	6.9 (42)	6.9 (6)	1	10.7 (41)	2.2 (7)	< 0.0001
*An. moucheti*	2.6 (16)	1.1 (1)	0.7097	2.6 (10)	2.2 (7)	0.8095
Total	612	87		384	315	
Distribution of *An. gambiae s.l.* sibling species % (n)						
*An; gambiae s.s*	76.9 (426)	92.5 (74)	0.0007	76.6 (255)	81.4 (245)	0.145
*An. coluzzii*	17.3 (96)	2.5 (2)	0.0002	16.5 (55)	14.3 (43)	0.444
*An. arabiensis*	5.8 (32)	5.0 (4)	1	6.9 (23)	4.3 (13)	0.1729

### Infection rate of Plasmodium spp. in abdomens and heads/thorax within the Anopheline family

**Fig. 2:**
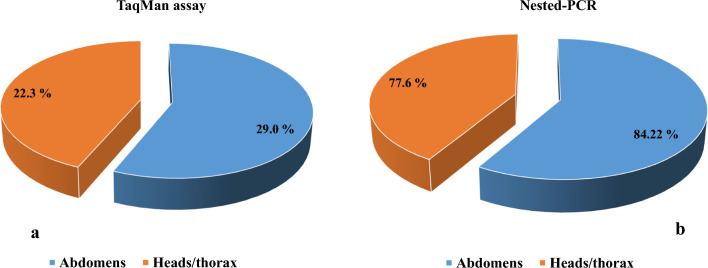
Proportion of the infection rate in abdomens and heaads/thorax: **a** by TaqMan assay and **b** by Nested-PCR

**Fig. 3:**
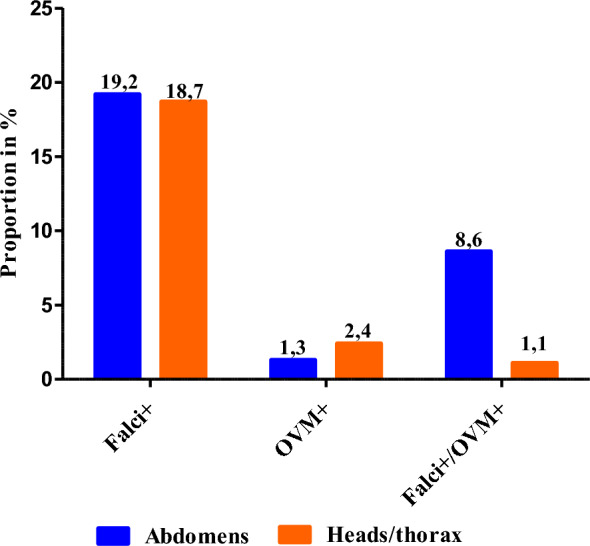
Detection of infection rate of the *Plasmodium spp* in Abdomens and Heads/Thorax of *Anopheles* by TaqMan assay

Out of the 699 *Anopheles* mosquitoes captured in this study, the *Plasmodium* species infection rate was higher in abdomens (29.0%; 203/699) compared to the heads/thorax (22.3% (156/699), when using TaqMan diagnostic technic (Fig. 2A). Overall, it emerges that of the 699 infected *Anopheles* mosquito abdomens, 19.2% (134/699) were found to be infected with *P. falciparum*, 1.3% (9/699) for infection with *OVM* + (*P. malariae, P. ovale* or *P. vivax*) and 8.6% (60/699) for mixed infection (*P. falciparum* with non-falciparum species: *P. malariae, P. ovale* or *P. vivax*). Moreover, of the 699 heads/thoraxes positive by TaqMan, 18.7% (131/699) were infected with *P. falciparum*, 2.4% (17/699) for OVM + and 1.1% (8/156) for mixed infection (Fig. 3). In the 203 abdomens and 156 heads/thorax infected by *Plasmodium* species using TaqMan method, only 84,2% (171/203) abdomens and 77.6% (121/156) were confirmed and discriminated in nested-PCR (Fig. 2B).

*Plasmodium* species in mono-infection identified in infected oocysts (abdomens) were predominated for *P. falciparum* 51.7% (105/203), following by *P. ovale* 2.0% (4/203) and *P. malariae* 1.5% (3/203). The co-infection in the oocysts (abdomens) were 17.3%, 6.4% and 1.0% for *P. falciparum/P. malariae, P. falciparum/P. ovale* and *P. malariae/P. ovale* co-infection, respectively. The triple infection (*P. falciparum/P. malariae/P. ovale*) oocysts infected *Anopheles* mosquitoes was at 3.6%. The identification of the sporozoite (heads/thorax) of *Plasmodium* species in the infecting mosquitoes showed that *P. falciparum* (66.0%) was the predominant transmitted parasites species, followed by *P. malariae* (10.9%) and *P. ovale* (6.4%). The prevalence of the sporozoite mono-infection by the three species (Table 2) was 62.2% (*P. falciparum*), 7.1% (*P. malariae*) and 3.2% for *P. ovale,* respectively. The overall prevalence of *Plasmodium* sporozoite co-infected mosquitoes was 1.0% (8/699), including 0.4% (3/699) (*P. falciparum/P. malariae*), and 0.3% (2/699) (for *P. falciparum/P. ovale or P. malariae/ P. ovale*). The triple (*P. falciparum/ P. malariae/ P. ovale*) sporozoite infected mosquitoes was 0.1% (1/699) (Table 3).

**Table 3:** Infection rate of *Plasmodium* species in abdomen and heads/thorax of *Anopheles*

*Plasmodium *spp.* infections (%)*	Abdomens (n = 203)	Head/thorax (n = 156)
Mono infection %(n)		
*P. falciparum*	51.7 (105)	62.2 (97)
*P. malariae*	1.5 (3)	7.1 (11)
*P. ovale*	2.0 (4)	3.2 (5)
Co-infection		
*P. falciparum/P. malariae*	17.3 (36)	1.9 (3)
*P. falciparum/P. ovale*	6.4 (13)	1.3 (2)
*P. malariae P. ovale*	1.0 (2)	1.3 (2)
*P. falciparum/P. malariae/P. ovale*	3.9 (8)	0.6 (1)

n: number of mosquitoes

### Infection rate of Plasmodium spp. according to the Anopheles species

**Fig. 4:**
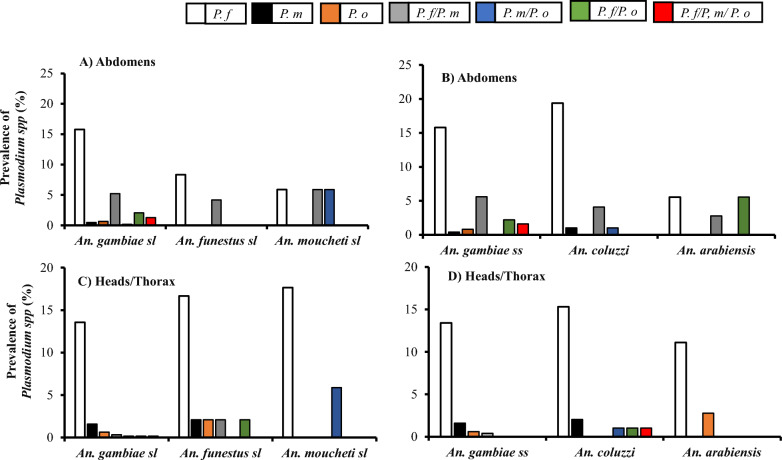
Prevalence of Plasmodium species infection in abdomens and Heads/thorax according to the *Anopheles* groups (**A**, **C**) and the Anopheles gambiae complex (**B**, **D**)

The distribution of the infection rate of oocyst and sporozoite of *Plasmodium* species was determined according to the *Anopheles* complex/groups (*An. gambiae s.l.*, *An. funestus* and *An. moucheti*) and Gambiae complex (*An. gambiae s.s.*, *An. coluzzii* and *An. arabiensis*) identified in this study (Fig. 4). Overall, regardless the type of infection (mono or co-infection), the sporozoites of all the *Plasmodium* species were detected in all the *Anopheles* species.

**Fig. 5:**
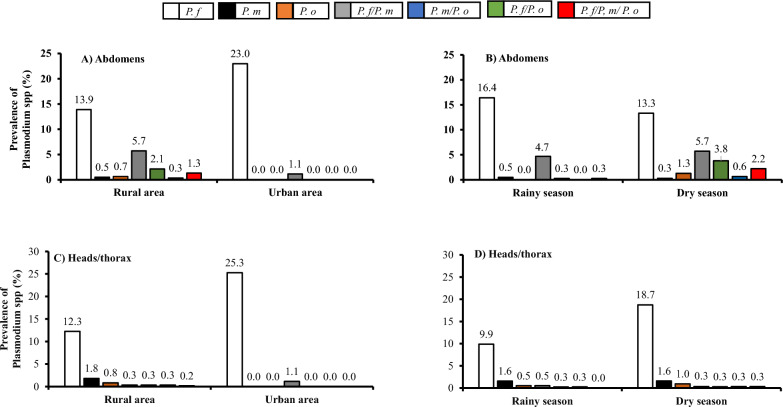
Prevalence of *Plasmodium* spp. infection in Anopheles species according to the study site (**A**, **C**) and the seasonality (**B**, **D**)

The overall infection rate of *Plasmodium* spp oocyst was predominant in *An. gambiae s.l.* (25.6%) followed by *An. moucheti* (17.6%) and *An. funestus* (12.5%). However, the reverse trend was observed with *Plasmodium* spp sporozoite infection, with *An. funestus* showing the higher prevalence (25.0%), followed by *An. moucheti* (23.5%) and then *An. gambiae s.l.* (16.6%). The prevalence of *P. falciparum* mono-infection in *Anopheles* was higher in both part: Abdomens (*An. gambiae s.l.*: 16.7%; *An. funestus*: 8.3% and *An. moucheti*: 5.9%) (Fig. 4A) and heads/thorax (*An. gambiae s.l.:* 17.2%; *An. funestus*: 16.7% and *An. moucheti*: 17.6%) (Fig. 5C) the mosquitoes. The rate of *P. malariae* and *P. ovale* sporozoite mono-infection was similar and higher in *An. funestus* (2.1% *P. malariae* and 2.1% *P ovale*) compared to *An. gambiae* (1.6% *P. malariae* and 0.6% *P. ovale*) and *An. moucheti* (0.0% *P. malariae* and 0.0% *P. ovale*). All types of sporozoite *Plasmodium* spp co-infection was found in *An. gambiae* (0.3% *P. falciparum/ P. malariae;* and 0.2% *P. falciparum/ P. ovale* and 0.2% *P. malariae/ P. ovale*), while only *P. falciparum* co-infection with either *P. malariae* or *P. ovale* was found in *An. funestus* with the same proportion (2.1%). A unique and high prevalence of *P. malariae/ P. ovale* (5.9%) sporozoite co-infection was detected in *An. moucheti*. The triple *P. falciparum/ P. malariae/ P. ovale* sporozoite infection was only detected in *An. gambiae s.l.* (0.2%).

Among the *An. gambiae s.l*., the overall prevalence of *Plasmodium* spp oocyst was predominant in *An. gambiae s.s.* (26.4%), followed by *An. coluzzii* (25.5%) and *An. arabiensis* (13.9%). However, the contrary was observed with *Plasmodium* spp sporozoite infection, where *An. coluzzii* (20.4%) had the higher rate of infection, followed by *An. gambiae ss* (16.0%) and then *An. arabiensis* (13.9%). The prevalence of *Plasmodium* spp mono-infection in *Anopheles gambiae* complex was predominated by *P. falciparum* both in abdomens (Fig. 4B) and heads/thorax (Fig. 4D), with *An. coluzzii* (abdomens: 19.4%; and heads/thorax: 15.3%) having the higher rate of infection compared to *An. gambiae s.s.* (abdomens: 15.8%; and heads/thorax: 13.4%) and *An. arabiensis* (abdomens: 5.6%; and heads/thorax: 11.1%). When both *P. malariae* and *P. ovale* sporozoite mono-infection were detected in *An. gambiae s.s.* (1.6% and 0.6%, respectively), only *An. coluzzii* and *An. arabiensis* were positive for only *P. malariae* (2.0%) and *P. ovale* (2.8%) sporozoite mono-infection respectively. The sporozoite co-infection with *P. malariae/ P. falciparum* (0.4%) was only detected in *An. gambiae s.s.*, while *An. coluzzii* showed similar prevalence of sporozoite co-infection with *ovale/P. falciparum* (1.0%) and *P. ovale/ P. malariae* (1.0%). No mixed sporozoite infection was detected in *An. arabiensis*. The prevalence of triple *P. falciparum/ P. malariae/ P. ovale* sporozoite infection was 1.0% and detected in *An. coluzzii*.

### Prevalence of Plasmodium spp. infection in Anopheles species according to the study site and the season

The rate of oocyst and sporozoite for *Plasmodium* species was determine according two ecological parameter including the municipally (Fig. 5A and C) and the season (Fig. 5B and D) in this study. *Plasmodium falciparum* infection was predominant in both abdomens and heads/thorax for all the study sites. The overall sporozoite infection rate of *Plasmodium* species was significantly higher in urban area (26.4%) compared to the rural area (16.0%) (p = 0.0162), although the total rate of oocyst infection was almost similar in rural (24.5%) and urban areas (24.1%). Only *P. falciparum* mono-infection (abdomens: 23.0%; heads/thorax: 25.3%) and dual *P. falciparum*/ *P. malariae* infection (abdomens: 1.1%; heads/thorax: 1.1%) were found in urban area. However, all the type of infection with *P. falciparum*, *P. malariae* and *P. ovale* were detected in rural setting with the predominance of sporozoite mono-infection of *P. falciparum* (12.3%), followed by *P. malariae* (1.8%) and *P. ovale* (0.8%).

The distribution of the *Plasmodium* spp infection with respect to the season (Fig. 5B and D) showed that, the overall prevalence of *Plasmodium* spp infection was significantly (p = 0.0009) higher during dry season compared to the rainy season for both in abdomens (dry: 27.3%; rainy: 22.1%) and heads/thorax (dry: 22.5%; rainy: 13.0%) of the mosquitoes. The rate of *P. falciparum* sporozoite mono-infection was predominant during raining and dry seasons (Fig. 5D), with a significant higher prevalence observed during the dry season (18.7%) compared to the rainy season (9.9%) (p = 0.0008). The same trend was observed with the prevalence of *P. ovale* sporozoite mono-infection (dry: 1.0% vs raining season: 0.5%), while the rate of *P. malariae* sporozoite mono-infection (1.6%) did not change with season. The prevalence of sporozoite co-infection with *P. falciparum*/ *P. malariae* during rainy season (0.5%) was higher than that observed during the dry season (0.3%). However, 0.3% of *Anopheles* had *P. ovale* sporozoite co-infection with either *P. falciparum* or *P. malariae* during both raining and dry seasons. The *P. falciparum*/ *P. malariae/ P. ovale* triple-infection was 0.3% during dry season.

### Entomological index of malaria transmission

Some entomological parameters were determined to assess the transmission of malaria in rural and urban areas. The results of the entomological index according to the study site and seasonality are reported in Table 3. The *Anopheles* man biting rate (ma) in the household of in Goma Tsé-Tsé district (rural area) was 0.84 bites/night (b/n), compared to 0.36 b/n obtained in the household from Madibou (urban) district. However, overall entomological inoculation rate (EIR) was 0.13 ib/p/n or 47.5 ib/p/y in the study setting. The EIR was 0.17 ib/p/n or 62.1 ib/p/year in rural area compared to 0.092 ib/p/n or 33.6 ib/p/y in urban area. This index was 0.18 ib/p/n during the dry season compared to 0.14 ib/p/n during the rainy season, and ma in the household remained constant during the raining (0.69) and dry (0.72) seasons. The ma of *An. gambiae* s*.l.* decreased when moving from rural (0.77 b/n) to urban area (0.34 b/n), while the contrary was observed with the EIR (rural: 0.15 ib/p/n vs 0.34 ib/p/n) of this species. However, the two parameters (ma and EIR) of *An. funestus s.l.* and *An. moucheti* were higher in rural area compared to the urban area as shown in Table 3. In addition, ma and EIR of *An. funestus s.l.* and *An. moucheti* were higher in raining season compared to dry season while no change in terms of ma and EIR was observed with season for *An. gambiae s.l.*

## Discussion

The present study aimed to determine the entomological indicators of *Plasmodium* spp transmission in Goma Tsé-Tsé and Madibou districts in the Republic of Congo. The entomological monitoring carried out revealed that *An. gambiae s.l.* was the major species in Goma Tsé-Tsé and Madibou districts, since it represents more than 90% of the total *Anopheline* caught in this study. The dominance of this species could be explained by its status as the main vector in the transmission of malaria in the Republic of Congo. These results are in line with previous studies in Cameroon [28, 29], in DRC [30] and in Gabon [31], that also reported the *An. gambiae s.l.* as the major group of *Anopheles* [2].

*Anopheles funestus s.l.* and *An. moucheti* were the other malaria vector identified in this study, but at the lower proportions. This study further confirms the presence of *An. funestus* and *An. moucheti* in Goma Tsé-Tsé district [18, 32], and reports for the first time the presence of these two species in a urban area in the Madibou district located at the southern part of Brazzaville. Like previous observation in southern areas of Cameroon [28], the proportion of caught *An. gambiae s.l.* was higher during the dry season compared to the wet season. However, the reverse distribution was observed with *An. funestus s.l.* between the two seasons. It is well known that Sahelian anopheline populations are seasonal, peaking in the rainy season [33, 34], as it was observed here with *An. funestus s.l..* The presence of a dry-season population of *An. gambiae s.l.* in this study reflects and responds to the presence of enduring larval habitat in the two localities. In fact, the sites where the study was carried out are characterized by the presence of neglected ponds and the culture gardening that contribute to the development of mosquitoes during dry season.

Molecular analyses showed that *An. gambiae s.s.* was the major species of the *An. gambiae complex*, followed by *An. coluzzii*, and *An. arabiensis*. These findings are in line with previous reports showing that *An. gambiae* ss is the predominant species within the *An. gambiae* complex in Central Africa [28, 35, 36]. The same species were also recently identified in western Burkina Faso [33], but with *An. arabiensis* being the major malaria vector*.*

In this study, *An. gambiae s.s.* was highly prevalent in urban area compared to the rural areas, while the contrary was observed with *An. coluzzii* between the two areas. The abundance of *An. gambiae s.s.* in Madibou district, which is the urban areas might be justify by the presence of active gardening cultures and the Djoué river bordering the area. The same observation was done in Benin. This is in line with previous studies conducted in Senegal which reported the abundance of *An. gambiae s.s.* in the locality bordered by Gambia River and where agriculture is active [37].

Out of the 699 *Anopheles* mosquitoes captured in this study, the *Plasmodium* spp infection rate was higher in abdomens compared to the heads/thorax compartment. This disparity is in line with that reported by previous studies [27, 38], suggesting the likely effect of mosquito immune response against the parasite development from the oocyst (abdomen) to the sporozoite (heads/thorax) stages [39, 40]. The mono-infection rate of *P. falciparum, P. malariae* and *P. ovale* was higher in heads/thorax compared to abdomens compartments. This could be explained by the competition between the different *Plasmodium* parasites by eliminating others when the parasite density of the latter is low in order to migrate towards the salivary gland of *Anopheles* mosquitoes [41]. The overall infection rate of *Plasmodium* spp sporozoite in the field-caught *Anopheles* was 77.6%, with *P. falciparum* being the predominant parasites species, followed by *P. malariae* and *P. ovale*. This trend supports the findings from a previous epidemiological study conducted during the same period in the same locality [13], confirming the local transmission of these malaria parasite in human population. The results obtained this study can be explained by the fact that *P. falciparum* is the predominant malaria parasite in the Republic of Congo, although control and elimination efforts should not ignore *P. malariae* and *P. ovale*, present in low infection rate [13]. These three *Plasmodium* species were also identified in malaria vectors in the centre region of Ivory Coast [42]. Previous study in Cameroon and Gabon also identified *P. falciparum* in malaria vectors, but did not specify the non-falciparum species due to the limitations of technic used [27, 43].

Among the *Anopheles* vectors, the infection rate of *Plasmodium* spp sporozoite was predominant in *An. funestus s.l.,* followed by *An. moucheti*, and then *An. gambiae s.l.* This result does not directly imply that *An. funestus* and *An. moucheti* are the major malaria vectors in these localities, since these two species represented less than 10% of the total field-caught *Anopheles*. The high infection rate of *Plasmodium* spp observed in *An. gambiae s.l.*, which was the major *Anopheles* species in this study confirm its first place in malaria transmission in central Africa [28–31]. These observations showed that most cases of malaria occurring in the studies sites are carried by *An. gambiae s.s.*, but *An. funestus* is the most competent species in the malaria transmission although the latter has been captured in small numbers 6.9% (48/699). The vectorial capacity of *An. funestus* has been shown in several regions of Cameroon [44, 45], so far, where it has been reported, it can sustain very high levels of malaria transmission [21, 45]. However, the identification of *An. funestus* and *An. moucheti* like in others countries [3, 7, 43, 44], suggests a deep study of the diversity of malaria transmission vectors in southern of Brazzaville.

*Plasmodium falciparum* parasite sporozoite was detected in all field-caught malaria vector in this study. *An gambiae s.l.* was responsible of the transmission of all *Plasmodium* species either in mono or in mix-infection, since all *Plasmodium* species sporozoite were detected in this vector. *Anopheles funestus* was showed to be able to transmit *P. falciparum* either in mono or in co-infection with *P. malariae* or *P. ovale*. However, a high rate of *P. malariae/ P. ovale* sporozoite co-infection was only detected in *An. moucheti*. All these findings suggest that there is a likely genetic susceptibility to *Plasmodium* species infection, as well as a competitive parasite interaction in case of co-infection in the *Anopheles* species. The presence of *Plasmodium* species in *Anopheles* vectors showed the participation of those species in malaria transmission.

It is well known that seasonality and the endemic locality are main factors related to epidemiology [13] as well as the level of malaria transmission [13]. The infection rate of *Plasmodium* species was also assessed according two ecological parameter including study site and the seasonality in the present work. The overall infection rate of *Plasmodium* spp sporozoite as well as the entomological inoculation rate of malaria vectors were significantly higher in urban area compared to the rural area. These results are in contrast with the findings commonly reported, showing that the urbanization is associated with the decrease of malaria transmission level [7]. One of the reasons of the results observed in this study might be the diversity of the *Plasmodium* species circulating between the two areas. All types of mono and mixt-infection of *Plasmodium* species were found in rural area, while the urban area was characterized by the predominance of *P. falciparum* mono-infection and dual *P. falciparum*/ *P. malariae* infection. Thus, the competitive development of two parasite species within a single mosquito might contribute to the decrease of the rate of infection.

According to the seasonality, the overall sporozoite infection rate and the entomological inoculation rate of *Anopheles* were higher during dry season compared to the wet season. The same observations were done during an entomological study conducted in the southern area of Cameroon [28]. However, these results are in discordance with the previous reported malaria epidemiology situation in the same study areas in Republic of Congo [13], as well as seasonal malaria vector and transmission dynamics reported in western Burkina-Faso [33]. All these findings may suggest a change of mosquito’s behaviour vis-a-vis the inhabitants that might occur between the two seasons, and according to the endemic area concerned in sub-Saharan Africa. Meanwhile, the fact that transmission occurs both during the dry and wet seasons in this study might be indicative of a perennial pattern in the locality. *Anopheles gambiae s.l*. was the most aggressive species with a bite rate higher than the other vectors, and *P. falciparum* being the predominant species*,* in the two area and during the rainy dry season. This is not improbable and is in support of previous studies in central Africa [28–30]. The annual average entomological inoculation rate (47.5 ib/p/y) recorded in this study was one of the highest ever recorded in the Republic of Congo and should be associated to a high infection rate in humans populations living in those areas [13]. Indeed, Trape & Zoulani [32] reported that annual EIRs of 22.5 ib/p/y are regularly associated with a prevalence of *Plasmodium* spp in humans [13], stand the risk of getting a daily infective mosquito bite of 0.18 ib/p/n during the dry season and 0.14 ib/p/n during the rainy season. In the previous study reported in malaria epidemiology situation [13], showed the high prevalence of malaria in dry season. Although the average annual entomological inoculation recorded in this study was lower than that in Gabon, the former suggest a *P. falciparum* prevalence in humans [27, 43].

## Conclusion

These findings highlight that malaria transmission remains high in rural and urban area in the south of Republic of Congo despite the ongoing control efforts, thereby indicating the need for more robust interventions. The vectors found in rural and urban setting mostly belong to the *An. gambiae *s.l. (*An. gambiae, An. coluzzii, An. arabiensis*), but with variable relative proportion from one locality to another.

## Data Availability

All data are fully available without restriction. Data are available from the FCRM Institutional Data Access. All request for Data should be addressed to the Executive Director of FCRM reachable by the following address Prof. Francine Ntoumi, Villa D6, Cité OMS-Djoué, Brazzaville, République du.

## Abbreviations

CeRMI: Centre de recherche sur les maladies infectieuses—Christoph Merieux
LBDEA: Laboratoire de la Biodiversité et Ecologie Animales
FST: Faculté des Sciences et Techniques
UMNG: Université Marien Ngouabi
EIR: Entomological Inoculation Rate
ib/p/n: Infectious bites/person/night
ib/p/y: Infectious bites/person/year

## References

[1] WHO. World Malaria Report 2022. Geneva: World Health Organization; 2022. https://www.who.int/teams/global-malaria-programme/reports/world-malaria-report-2022.

[2] Fontenille D, Cohuet A, Awono-Ambene P, Antonio-Nkondjio C, Wondji C, Kengne P (2003). Systématique et biologie des anophèles vecteurs de *Plasmodium* en Afrique, données récentes. Med Trop (Mars).

[3] Antonio-Nkondjio C, Kerah CH, Simard F, Awono-Ambene P, Chouaibou M, Tchuinkam T (2006). Complexity of the malaria vectorial system in Cameroon: contribution of secondary vectors to malaria transmission. J Med Entomol.

[4] Keiser J, Utzinger J, de Castro M, Smith TA, Tanner M, Singer BH (2004). Urbanization in sub-saharan Africa and implication for malaria control. Am J Trop Med Hyg..

[5] Hiwat H, Bretas G (2011). Ecology of *Anopheles darlingi* Root with respect to vector importance: a review. Parasit Vectors.

[6] Amvongo-Adjia N, Wirsiy EL, Riveron JM, Ndongmo WPC, Enyong PA, Njiokou F (2018). Bionomics and vectorial role of anophelines in wetlands along the volcanic chain of Cameroon. Parasit Vectors.

[7] Bigoga JD, Manga L, Titanji VPK, Coetzee M, Leke RFG (2007). Malaria vectors and transmission dynamics in coastal south-western Cameroon. Malar J.

[8] Doumbe-Belisse P, Kopya E, Ngadjeu CS, Sonhafouo-Chiana N, Talipouo A, Djamouko-Djonkam L (2021). Urban malaria in sub-Saharan Africa: dynamic of the vectorial system and the entomological inoculation rate. Malar J.

[9] Mouchet J, Carnevale P, Coosemans M, Julvez J, Manguin S, Richard-Lenoble D (2004). Biodiversité du paludisme dans le monde.

[10] Bayoh MN, Thomas CJ, Lindsay SW (2001). Mapping distributions of chromosomal forms of *Anopheles gambiae* in West Africa using climate data. Med Vet Entomol.

[11] Ortiz DI, Piche-Ovares M, Romero-Vega LM, Wagman J, Troyo A (2022). The impact of deforestation, urbanization, and changing land use patterns on the ecology of mosquito and tick-borne diseases in Central America. Insects.

[12] Programme National Lutte contre Paludisme (PNLP). Rapport d'activités. Ministère de la santé et de la population, République du Congo (Congo-Brazzaville); 2020.

[13] Mbama Ntabi JD, Lissom A, Djontu JC, Diafouka-Kietela S, Vouvoungui C, Boumpoutou RK (2022). Prevalence of non-*Plasmodium falciparum* species in southern districts of Brazzaville in The Republic of the Congo. Parasit Vectors.

[14] Tsumori Y, Ndounga M, Sunahara T, Hayashida N, Inoue M, Nakazawa S (2011). *Plasmodium falciparum*: differential selection of drug resistance alleles in contiguous urban and peri-urban areas of Brazzaville. Republic of Congo PLoS One.

[15] Adam JP. Répartition géographique des Anophèles en République du congo (Brazzaville). Office de la recherche scientifique et technique outre-mer. 1957.

[16] Carnevale P, Bosseno MF, Zoulani A, Miche LR, Molez JF. La dynamique de la transmission du paludisme humain en zone de savane herbeuse et de forêt dégradée des environs nord et sud de Brazzaville, R.P. du Congo. Cahiers Orstom, sér Ent Méd Parasitol 1985;95–115.

[17] Geography of the Republic of Congo [http://fr.getamap.net/about/republic_of_the_congo/geography.html]

[18] Nianga BGOT, Bitsindou P, Lenga A (2019). Impact of the use of mosquito nets on malaria transmission in Djoumouna (Brazzaville-congo). Int J Adv Res.

[19] Gillies MT, De Meillon B. The Anophelinae of Africa south of the Sahara (Ethiopian zoogeographical region). Publ South Afr Inst Med Res. 1968.

[20] Gillies M, Coetzee M (1987). A supplement to the Anophelinae of Africa south of the Sahara (Afrotropical region). Publ South Afr Inst Med Res.

[21] Nkemngo FN, Mugenzi LMJ, Terence E, Niang A, Wondji MJ, Tchoupo M (2020). Multiple insecticide resistance and *Plasmodium* infection in the principal malaria vectors *Anopheles funestus* and *Anopheles gambiae* in a forested locality close to the Yaoundé airport. Cameroon Wellcome Open Res.

[22] Livak KJ (1984). Organization and mapping of a sequence on the *Drosophila melanogaster* X and Y chromosomes that is transcribed during spermatogenesis. Genetics.

[23] Santolamazza F, Mancini E, Simard F, Qi Y, Tu Z, della Torre A. (2008). Insertion polymorphisms of SINE200 retrotransposons within speciation islands of *Anopheles gambiae* molecular forms. Malar J.

[24] Koekemoer LL, Kamau L, Hunt RH, Coetzee M (2002). A cocktail polymerase chain reaction assay to identify membersof the *Anopheles funestus* (Diptera: Culicidae) group. Am J Trop Med Hyg.

[25] Bass C, Nikou D, Blagborough AM, Vontas J, Sinden R, Williamson MS (2008). PCR-based detection of *Plasmodium* in *Anopheles* mosquitoes: a comparison of a new high-throughput assay with existing methods. Malar J..

[26] Boonma P, Christensen PR, Suwanarusk R, Price RN, Russell R, Lek-Uthai U (2007). Comparison of three molecular methods for the detection and speciation of *Plasmodium vivax* and *Plasmodium falciparum*. Malar J.

[27] Nkemngo FN, Mugenzi LMJ, Tchouakui M, Nguiffo-Nguete D, Wondji MJ, Mbakam B (2022). Xeno-monitoring of molecular drivers of artemisinin and partner drug resistance in *P. falciparum* populations in malaria vectors across Cameroon. Gene..

[28] Bigoga JD, Nanfack FM, Awono-Ambene PH, Patchoké S, Atangana J, Otia VS (2015). Seasonal prevalence of malaria vectors and entomological inoculation rates in the rubber cultivated area of Niete South Region of Cameroon. Parasit Vectors..

[29] Manga L, Bouchite B, Toto JC, Froment A (1997). [Anopheles species and the transmission of malaria in the forest/savannah transition zone in central Cameroon] (in French). Bull Soc Pathol Exot.

[30] Matubi EM, Bukaka E, Luemba TB, Situakibanza H, Sangaré I, Mesia G (2015). Détermination des paramètres bioécologiques et entomologiques d’Anopheles gambiae s.l. dans la transmission du paludisme à Bandundu-ville, République Démocratique de Congo. Pan Afr Med J..

[31] Sylla EH, Kun JF, Kremsner PG (2000). Mosquito distribution and entomological inoculation rates in three malaria-endemic areas in Gabon. Trans R Soc Trop Med Hyg.

[32] Trape JF, Zoulani A (1987). Malaria and urbanization in central Africa: the example of Brazzaville. Part II: Results of entomological surveys and epidemiological analysis. Trans R Soc Trop Med Hyg..

[33] Epopa PS, Collins CM, North A, Millogo AA, Benedict MQ, Tripet F (2019). Seasonal malaria vector and transmission dynamics in western Burkina Faso. Malar J.

[34] Lehmann T, Dao A, Yaro AS, Diallo M, Timbiné S, Huestis DL (2014). Seasonal variation in spatial distributions of *Anopheles gambiae* in a Sahelian village: evidence for aestivation. J Med Entomol..

[35] Atangana J, Fondjo E, Fomena A, Tamesse JL, Patchoké S, Ndjemaiuml HN (2009). Seasonal variations of malaria transmission in Western Cameroon highlands: entomological, parasitological and clinical investigations. J Cell and Animal Biol.

[36] Mourou JR, Coffinet T, Jarjaval F, Cotteaux C, Pradines E, Godefroy L (2012). Malaria transmission in Libreville: results of a one year survey. Malar J.

[37] Caputo B, Nwakanma D, Jawara M, Adiamoh M, Dia I, Konate L (2008). Anopheles gambiae complex along The Gambia river, with particular reference to the molecular forms of *An. gambiae* s.s. Malar J..

[38] Menze BD, Wondji MJ, Tchapga W, Tchoupo M, Riveron JM, Wondji CS (2018). Bionomics and insecticides resistance profiling of malaria vectors at a selected site for experimental hut trials in central Cameroon. Malar J.

[39] Clayton AM, Dong D, Dimopoulos G (2014). The *Anopheles* innate immune system in the defense against malaria infection. J Innate Immun.

[40] Smith RC, Barillas-Mury C (2016). *Plasmodium* oocysts: overlooked targets of mosquito immunity. Trends Parasitol.

[41] Hume JC, Tunnicliff M, Ranford-Cartwright LC, Day KP (2007). Susceptibility of *Anopheles gambiae* and *Anopheles stephensi* to tropical isolates of *Plasmodium falciparum*. Malar J.

[42] Zoh DD, Yapi A, Adja MA, Guindo-Coulibaly N, Kpan DM, Sagna AB (2020). Role of *Anopheles gambiae* s.s. and *Anopheles coluzzii* (Diptera: Culicidae) in human malaria transmission in rural areas of Bouaké, in Côte d’Ivoire. J Med Entomol..

[43] Boussougou-Sambe ST, Woldearegai TG, Doumba-Ndalembouly AG, Ngossanga B, Mba RB (2022). Assessment of malaria transmission intensity and insecticide resistance mechanisms in three rural areas of the Moyen Ogooué Province of Gabon. Parasit Vectors.

[44] Highlights on *Anopheles nili* and *Anopheles moucheti*, malaria vectors in Africa. In: *Anopheles* mosquitoes: new insights into malaria vectors. Manguin S (ed.). InTechOpen, 2013. 28045480

[45] Djamouko-Djonkam L, Nkahe DL, Kopya E, Talipouo A, Ngadjeu CS, Doumbe-Belisse P (2020). Implication of *Anopheles funestus* in malaria transmission in the city of Yaoundé. Cameroon Parasite.

